# Classroom innovation atmosphere and student creativity: the roles of AI technology application, intrinsic motivation, and self-efficacy

**DOI:** 10.3389/fpsyg.2025.1735425

**Published:** 2025-12-10

**Authors:** Chao Jiang, Mengjie Han, Zhangwei Mao, Jing Yu, Fei Jiang, Yuhao Liang

**Affiliations:** 1 Zhejiang University of Finance and Economics Dongfang College, Haining, China; 2 School of Education and English, University of Nottingham Ningbo China, Ningbo, China; 3 School of Business, Nanjing University, Nanjing, China; 4 College of International Education, Guizhou University, Guiyang, Guizhou, China

**Keywords:** classroom innovation atmosphere, creativity, AI technology application, self-efficacy, intrinsic motivation

## Abstract

Creativity is a key capability that drives innovation and solves complex problems, serving as an important pathway to realizing personal potential. Based on social cognitive theory, this study constructs a theoretical model with classroom innovation atmosphere as the independent variable, student creativity as the dependent variable, AI technology application and intrinsic motivation as mediating variables, and self-efficacy as the moderating variable. A survey was conducted among 270 college students from the Jiangsu-Zhejiang-Shanghai region, and Hayes’s PROCESS macro (Model 7) was employed to perform Bootstrap sampling (sample size = 5,000) for testing. The results show that: classroom innovation atmosphere has a significant positive impact on student creativity (*β* = 0.182, *p* < 0.01); AI technology application (indirect effect = 0.0267, 95% CI [0.0034, 0.0598]) and intrinsic motivation (indirect effect = 0.1029, 95% CI [0.0281, 0.1820]) play significant mediating roles between classroom innovation atmosphere and creativity; self-efficacy not only positively moderates the relationship between classroom innovation atmosphere and AI technology application (*β* = 0.339, *p* < 0.05) as well as intrinsic motivation (*β* = 0.083, *p* < 0.05), but also moderates the indirect effects mediated by AI technology application and intrinsic motivation (moderated mediation indices were 0.0373, 95% CI [0.0023, 0.1195] and 0.6574, 95% CI [0.2512, 0.8066], respectively). The findings enhance the understanding of the relationship between classroom innovation atmosphere and student creativity, providing theoretical foundations and practical insights for educators to foster student creativity.

## 1 Introduction

Driven by the global wave of innovation, cultivating talents with innovative thinking and practical abilities has become a core strategic directive for nations to seize future development opportunities and enhance comprehensive national strength. The Organisation for Economic Co-operation and Development (OECD) clearly states in its research report *Future of Education and Skills 2030* that, as digital transformation deepens, global education systems are facing unprecedented challenges and opportunities. The report advocates for countries to prioritize fostering students’ creativity, critical thinking, and collaboration skills as the core competencies of future education to adapt to an ever-changing global society. Multiple studies indicate that school classroom environments conducive to creative learning can improve students’ academic performance, learning motivation, and enthusiasm for participating in school activities, thereby fostering creative skills (Davies et al., 2013). As a breeding ground for student creativity, the innovative atmosphere in classrooms plays a significant role in cultivating individual creative thinking. A positive classroom innovation atmosphere can effectively stimulate students’ intrinsic motivation, ignite their passion for learning, enhance their focus during classroom activities, and increase their enthusiasm for participating in various school activities (Li and Li, 2025). Such an environment not only benefits academic improvement but also lays a solid foundation for developing students’ creative skills. The increasing prevalence of artificial intelligence (AI) technology is continuously transforming the traditional classroom ecosystem. In its report *Artificial Intelligence in Education: Challenges and Opportunities for Sustainable Development*, UNESCO analyzes the role of AI in education, highlighting how integrating it into classroom teaching not only optimizes instructional processes but also fosters an innovative atmosphere that encourages exploration and experimentation, thereby effectively enhancing students’ creativity. Some scholars advocate that schools should utilize AI tools like ChatGPT as teaching aids to unlock student creativity and provide personalized tutoring (Oranga et al., 2024). Advanced AI educational tools can play a significant role by collecting and deeply analyzing students’ learning data in real-time, delivering personalized feedback. This enables educators to overcome the limitations of traditional teacher-student understanding, allowing them to grasp each student’s learning progress and knowledge mastery more promptly, comprehensively, and thoroughly—thereby identifying latent learning potential (Zhang and Zhang, 2024). Through this approach, teachers can gain deeper insights into students’ needs, adjust teaching strategies accordingly, and create a more tailored, innovative classroom ecosystem that aligns with individual student development. Singapore, under its “Smart Nation” initiative, has integrated AI tools into its education system to support students in developing creative skills and thinking abilities. Similarly, the European Union, through its *Horizon 2020* program, has invested substantial resources to promote educational innovation, encouraging the extensive use of digital tools such as AI, big data, and virtual reality in classroom teaching. These efforts enhance teacher-student interaction and student engagement while providing technological support for personalized learning and educational equity. Establishing an active, open, and innovation-driven classroom environment, combined with AI integration, has become a widespread consensus in global education reform. Moreover, it provides critical environmental and institutional support for fostering and developing students’ creativity.

Although existing research has explored the impact of classroom environments, technology applications, and motivational factors on student creativity, most studies remain at the level of analyzing single or dual-variable relationships, lacking an integrated multi-path framework that combines “environment-technology-motivation-cognitive beliefs.” Particularly against the backdrop of AI technologies rapidly integrating into educational practices, the mediating and moderating mechanisms of how an innovative classroom atmosphere stimulates students’ use of AI tools and enhances their intrinsic motivation to boost creativity have yet to undergo systematic verification. This study constructs and validates a comprehensive model that treats AI technology application and intrinsic motivation as parallel mediating variables, with self-efficacy as a key moderating variable. Intrinsic motivation, driven by an individual’s interest, curiosity, pleasure, and willingness to engage in challenges, serves as a crucial condition for fueling creativity (Amabile, 1993). Self-efficacy, defined as an individual’s belief in their own capabilities, plays a pivotal role in the innovation process (Bandura, 1977). Students with high self-efficacy embrace challenges in innovative environments, whereas low self-efficacy may inhibit creative behaviors. The model not only reveals the dual-path mechanism through which an innovative classroom atmosphere influences student creativity but also demonstrates how students’ self-beliefs act as a “catalyst” in converting environmental support into technological practice and intrinsic motivation. This further deepens the understanding of the triadic reciprocal dynamics in social cognitive theory (Bandura, 1999) Zhao within the context of educational innovation, providing theoretical explanations and empirical evidence for cultivating creativity in the AI era.

This study is based on social cognitive theory (Bandura, 2001) to explore the influence mechanism of classroom innovation atmosphere on student creativity, further analyzing the mediating roles of AI technology application and intrinsic motivation, as well as the moderating effect of self-efficacy. The research seeks to address the following questions: Does student creativity benefit from classroom innovation atmosphere? Do AI technology application and intrinsic motivation mediate the relationship between classroom innovation atmosphere and creativity? How does self-efficacy moderate the relationships between classroom innovation atmosphere and AI technology application/intrinsic motivation? By addressing these questions, this study will provide empirical evidence for clarifying the role of classroom innovation atmosphere in fostering student creativity, offering theoretical support and practical solutions for educators to effectively utilize AI technology in creating an innovative classroom environment, thereby stimulating students’ intrinsic psychological motivation, enhancing their self-efficacy, and ultimately cultivating their creative abilities.

## 2 Theoretical foundations and research hypotheses

### 2.1 Theoretical framework

Bandura’s (2001) social cognitive theory emphasizes the interaction among individual cognition, behavior, and the environment, providing an integrative framework for understanding human behavior in specific contexts. According to this theory, this study regards classroom innovation atmosphere as a key environmental factor, defines AI technology application as specific technology usage behavior, and treats intrinsic motivation and self-efficacy as motivation-related cognitive and belief-related cognitive variables, respectively. Together, they form a “environment-cognition-behavior-outcome” causal chain to explore the formation mechanism of student creativity.

In social cognitive theory, behavior is not solely influenced by the environment but also regulated and driven by individual cognition. As a supportive environment, an innovative classroom atmosphere can reduce students’ perceived risks and barriers when adopting new technologies, encouraging them to view AI as a tool for exploration and creation. This, in turn, enhances their creative performance through technological empowerment. The environment indirectly affects behavior and outcomes by activating individuals’ cognitive processes. When the classroom atmosphere encourages free exploration, students are more likely to develop intrinsic interest in learning itself, leading to sustained engagement in creative activities. From an intrinsic perspective, the adoption of AI technology is an “instrumental behavior” that relies on individuals’ self-confidence in their abilities and the supportive forces of the environment. Social cognitive theory clearly indicates that individuals with high self-efficacy are more willing to translate environmental opportunities into actual actions, demonstrating stronger adaptability and persistence when facing new technologies (Van Dinther et al., 2011). Self-efficacy not only amplifies the positive effect of an innovative classroom atmosphere on AI technology adoption but also strengthens individual persistence in innovative environments, creating a reinforcing cycle of “environment-cognition-behavior.”

Social cognitive theory was ultimately adopted as the theoretical framework for this study because its triadic reciprocal determinism model can simultaneously encompass multi-level interactions among environment, cognition, and behavior. It effectively explains the mediating role of AI technology usage between innovation atmosphere and creativity—not merely as behavioral responses to environmental stimuli, but also as instrumental practical activities driven by cognitive beliefs. This theoretical choice enables the study to transcend traditional single-path analysis and reveal the cognitive-behavioral mechanisms of student creativity cultivation within a dynamic, integrated framework.

### 2.2 Definition and operationalization of classroom innovation atmosphere

Classroom innovation atmosphere refers to the multidimensional set of factors in the classroom environment that stimulate and support students’ creative thinking and behaviors. It emphasizes the classroom as a dynamic system, showcasing innovation-oriented characteristics in aspects such as teachers’ instructional performance, forms of student interaction, and the arrangement of teaching tasks. Its core components include supportive teacher behaviors, open communication and collaboration among students, and the design of challenging and open-ended learning tasks. Together, these elements create an environment where students feel psychologically secure, bold to experiment, and enthusiastic about exploring the unknown. Compared to the general concepts of “learning atmosphere” or “classroom environment,” classroom innovation atmosphere focuses more on the factors that directly promote innovative steps. Learning atmosphere typically covers broader academic, managerial, and emotional dimensions, whereas classroom innovation atmosphere refers to specific environmental conditions that inspire the generation of ideas, support unconventional thinking, and encourage practical exploration. Traditional classroom environments may emphasize discipline and knowledge transmission, while classroom innovation atmosphere highlights openness, exploration, and support.

In this study, we adopted and adapted the Classroom Environment Scale developed by Fraser. This scale measures multiple dimensions, such as communication atmosphere, teacher encouragement, and innovation support, allowing us to effectively quantify this construct and examine its relationship with student creativity.

### 2.3 Classroom innovation atmosphere and creativity

Creativity is defined as the ability to generate novel and useful ideas (Amabile et al., 2018), serving as a critical foundation for individuals, teams, and organizations to achieve innovation (Shalley et al., 2004). Research on creativity primarily unfolds from three perspectives: the individual perspective, the environmental perspective, and the interaction perspective (Lee and Lee, 2023). The individual perspective focuses on the effects of cognition, personality, and motivation on creativity; the environmental perspective examines how external conditions, cultural atmosphere, and social support facilitate creative thinking; the interaction perspective investigates the dynamic relationship between individuals and their environment, demonstrating their mutual influence on creativity development.

From the individual perspective, cognition and personality traits significantly impact creativity, with openness showing a positive correlation with creative ability (McCrae and Costa, 1997). Individual motivation is also a key factor influencing creativity—just as intrinsic motivation drives students who develop an interest in a subject to explore it deeply and innovate continuously.

The environmental perspective highlights the influence of external conditions on creativity formation. Existing research indicates that people’s work environments are closely linked to their creativity (Deng et al., 2016), and a supportive work environment can stimulate individuals’ creative potential (Amabile, 1997). In education, fostering student creativity is inseparable from the “environmental perspective” of classroom innovation. When teachers cultivate an inclusive classroom atmosphere, students are more likely to express their ideas freely, thereby activating creative thinking. Teachers can employ teaching strategies such as open-ended questions, teamwork, and creative activities to facilitate group discussions and brainstorming, effectively creating a challenging learning environment that encourages students to approach problems from diverse perspectives. This nurtures their ability for creative expression and enhances problem-solving skills (Collard and Looney, 2014).

The interaction perspective emphasizes the dynamic relationship between individuals and their environment. Creativity does not develop unidirectionally but emerges through mutual influence between individuals and their surroundings—individual creativity often flourishes when supported by conducive environments (Sawyer and Henriksen, 2023). Thus, students’ creative expressions in class are closely tied to classroom atmosphere. Based on prior theoretical and empirical research, this study proposes the hypothesis:

*H1*: Classroom innovation atmosphere significantly positively influences students’ creativity.

### 2.4 The mediating role of AI technology application and intrinsic motivation

The application scope of AI technology in education continues to expand, particularly in higher education, where artificial intelligence has been introduced into classroom activities. This not only provides students with new learning tools but also serves as a crucial enabling method for fostering student creativity. From a theoretical mechanism perspective, AI technology applications primarily enhance creativity through three pathways: cognitive offloading, cognitive augmentation, and supporting exploration in novel idea spaces. Cognitive offloading refers to AI’s ability to automate tedious tasks such as information gathering and data computation, thereby freeing students’ cognitive resources from routine, low-level mental burdens and enabling them to focus more on high-level creative planning and critical thinking. Cognitive augmentation means AI tools can provide information, perspectives, and analytical frameworks that surpass an individual’s prior knowledge base, thereby expanding students’ cognitive boundaries and creating favorable conditions for cross-disciplinary associations and knowledge integration. Supporting exploration in novel idea spaces manifests as AI’s capability to rapidly generate multiple alternative solutions and simulate outcomes under various scenarios, allowing students to conduct low-cost mental experimentation—thus encouraging bold trial-and-error and continuous optimization. These advantages are often difficult to achieve in traditional classroom settings due to time and resource constraints.

Creativity is a core element driving individual and societal progress, and high-quality educational environments form the foundation for cultivating such creativity (Csikszentmihalyi, 1996). Classrooms with a strong innovation atmosphere demonstrate significant effects in enhancing student creativity, while also encouraging active student participation and application of AI technologies. Teachers’ guidance plays an equally critical role—educators can create open learning environments that stimulate students’ thinking and experimentation, thereby facilitating easier adoption and utilization of AI technologies (Runco, 2014). For instance, teachers may employ AI tools to implement personalized instruction based on students’ interests and capabilities, which not only improves learning outcomes but also strengthens students’ creative thinking.

Students can also skillfully utilize AI technology to facilitate their creative thinking activities more smoothly. For instance, by using AI for data analysis and situational exploration, students can more swiftly and efficiently uncover the essence of complex problems. With AI’s assistance in generating multiple potential solutions, they can explore a broader idea space and ultimately develop innovative responses. AI technology further assists students in conducting self-reflection and evaluation of their learning, thereby promoting self-improvement. Effective application of AI technology not only relies on classroom innovation atmosphere but can reciprocally enhance this atmosphere’s vitality. During learning processes, AI provides real-time feedback and personalized learning suggestions, encouraging more active student engagement in creative tasks (Deci and Ryan, 2000). It transcends being merely a tool, serving instead as a powerful support for classroom innovation and creativity enhancement.

Artificial intelligence is a broad discipline encompassing multiple branches, each with unique research directions and application scenarios. In terms of its role in the creative process, AI technology in education can generally be divided into two categories: generative AI, which autonomously produces entirely new content such as text, code, and images, directly participating in the ideation phase; and instrumental AI, which primarily leverages functions like data analysis, intelligent tutoring, and process optimization to support students’ exploration and practice, facilitating the implementation phase of creativity. This study primarily focuses on instrumental AI technology—applications that serve as aids to help students more efficiently gather information, analyze data, refine solutions, and execute tasks. Examples include intelligent learning systems, data mining tools, and adaptive teaching platforms. The key role of these technologies lies in providing a foundation for deeper creative thinking and exploration through “cognitive offloading” and “cognitive extension,” which aligns more closely with the theoretical framework this study seeks to explore—leveraging environmental support to activate individuals’ intrinsic agency. Based on this analysis, we propose the following hypothesis:

*H2*: AI technology application plays a mediating role between classroom innovation atmosphere and creativity.

Intrinsic motivation refers to the motivation generated by an individual’s interest in the learning activity itself during the learning process. Unlike extrinsic motivation, intrinsic motivation emphasizes the emotional satisfaction and sense of achievement brought by the learning activity itself. A classroom innovation environment that encourages free thinking and maintains a lively atmosphere is more likely to stimulate students’ intrinsic motivation, thereby promoting greater engagement in learning (Fan and Cai, 2022).

When students perceive learning activities as both challenging and interesting, they are more likely to invest time and effort into learning to obtain a sense of fulfillment. Teachers can employ teaching methods such as open-ended questions and cooperative learning to create an innovative classroom atmosphere, inspiring students to engage in deep thinking and discussion while experiencing the joy of learning during exploration. Meanwhile, timely encouragement from teachers is essential, as these behaviors help students recognize their continuous progress, thereby motivating them to continue exploring and seeking knowledge. Intrinsic motivation is also a key factor influencing students’ creativity levels, with existing research confirming the positive relationship between intrinsic motivation and creativity (Ryan and Deci, 2000). Zhang and Bartol’s (2010) study demonstrates that intrinsic motivation promotes creative performance by influencing individuals’ engagement in the creative process. Based on the above analysis, this study proposes the following hypothesis:

*H3*: Students’ intrinsic motivation plays a mediating role between classroom innovation atmosphere and creativity.

### 2.5 The moderating role of self-efficacy

Self-efficacy refers to an individual’s belief in their own capabilities, a concept derived from Albert Bandura’s social cognitive theory (Bandura, 2000). Bandura posited that such beliefs not only influence people’s choice of behaviors but also affect their persistence levels and effort investment when facing challenges (Bandura, 2001). In educational contexts, this belief is particularly crucial as it directly impacts students’ learning motivation and strategies, ultimately affecting their academic outcomes. According to social cognitive theory, environmental influences on individual behavior and internal states do not uniformly affect everyone but are significantly modulated by individual cognitive factors. A classroom environment that fosters innovation provides opportunities and possibilities, but whether and how individuals utilize these environmental resources depends on their beliefs about their own capabilities. Treating self-efficacy as a moderating variable is key to transitioning from an “environment-driven” model to an “individual-environment interaction” model, which is essential for understanding the mechanisms of innovative classroom atmospheres.

Self-efficacy serves as a key psychological variable that moderates intrinsic motivation by influencing individuals’ cognitive processes and emotional responses (Schunk, 2003). Students with stronger self-efficacy typically demonstrate higher intrinsic motivation when approaching learning tasks, exhibit greater enthusiasm for learning, and are more willing to challenge themselves and overcome difficulties, thereby gaining more sense of achievement and satisfaction during learning (Zimmerman, 2000). This positive mindset enables them to maintain higher academic focus and persevere when encountering complex problems. Particularly when introducing emerging technologies like AI, the process itself comes with uncertainties and challenges. Students with high self-efficacy are more likely to perceive the encouragement of technological application in an innovative classroom as a challenging opportunity. They are confident in their ability to master and effectively use AI tools (Pajares, 1996), thus more actively and boldly translating environmental support into concrete exploratory actions. In contrast, students with low self-efficacy may hesitate even in an innovation-encouraging environment due to doubts about their technical competence, making it difficult for them to effectively initiate the “environment-technology application-creativity” mediating pathway. The purpose of a classroom innovation atmosphere is to stimulate students’ intrinsic motivation, but this effect also relies on students’ self-beliefs. Students with high self-efficacy, when faced with open-ended, exploratory learning tasks, are more confident in enjoying the thrill of challenging themselves. The environment more readily activates and sustains their intrinsic motivation. However, for students with low self-efficacy, the same open-ended tasks may cause them to focus more on the possibility of failure rather than the intrinsic joy of exploration, making it harder for their intrinsic motivation to be strongly ignited.

Therefore, investigating how self-efficacy moderates the influence of classroom innovation atmosphere on AI technology adoption and individual intrinsic motivation holds significant implications for cultivating university students’ creativity in the digital era. Based on the above analysis, this study proposes the following hypotheses:

*H4a*: Self-efficacy has a significant positive moderating effect on the relationship between classroom innovation atmosphere and AI technology application;

*H4b*: Self-efficacy has a significant positive moderating effect on the relationship between classroom innovation atmosphere and students’ intrinsic motivation;

*H5a*: Self-efficacy positively moderates the mediating effect of AI technology application between classroom innovation atmosphere and student creativity;

*H5b*: Self-efficacy positively moderates the mediating effects of both student intrinsic motivation and AI technology application between classroom innovation atmosphere and student creativity.

**Figure 1:**
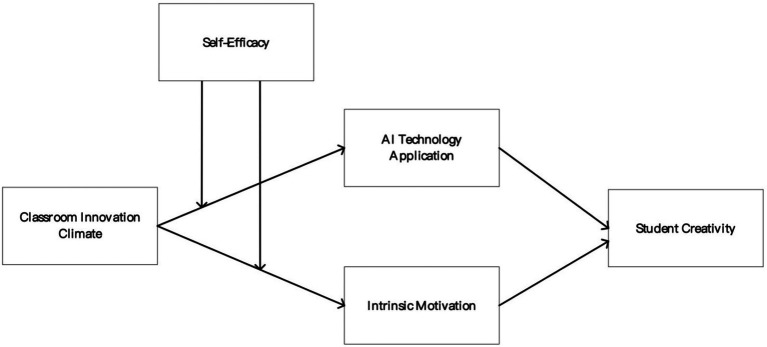
Theoretical model.

In summary, we have constructed the following theoretical model (as shown in Figure 1).

## 3 Research design

### 3.1 Sample and data collection

This study focuses on university students in the Jiangsu-Zhejiang-Shanghai region, collecting data through questionnaire surveys. This region is one of China’s most economically developed areas with the highest concentration of educational resources. Local universities generally rank at the forefront in terms of teaching and educational reforms, as well as the integration and application of information technology, demonstrating a high level of acceptance and proficiency in innovative teaching tools like AI technology. This provides a rich context for studying classroom innovation and the application of AI. The region has always prioritized cultivating innovative talent, with diverse and representative academic programs and teaching models across its universities, reflecting the current practices and challenges of creativity cultivation in Chinese higher education. Therefore, selecting university students from this region as research subjects enhances the relevance of the study sample and the insightful value of the research findings. Electronic questionnaires were distributed to first- through fourth-year students via online platforms across various universities, with data collection conducted in two phases spaced 1 month apart. To ensure consistency between the two phases, respondents were required to use “initials + last four digits of phone number” as identifiers for subsequent screening and matching. The first phase collected students’ basic information and classroom innovation capability data, distributing 420 questionnaires and obtaining 369 valid responses. The second phase gathered data on student creativity, AI technology application, intrinsic motivation, and self-efficacy, distributing 369 questionnaires and yielding 270 valid samples after removing invalid responses, representing a valid response rate of 73.2% (Table 1).

**Table 1:** Sample description (*N* = 270).

Category	Characteristics	Number	Percentage
Gender	Male	99	36.67%
	Female	171	63.33%
Grade level	Freshman	103	38.15%
	Sophomore year	84	31.11%
	Junior year	60	22.22%
	Senior year	23	8.52%
Location (Province)	Zhejiang	85	31.48%
	Shanghai	93	34.44%
	Jiangsu	92	34.07%
Major	Science and Engineering	62	22.96%
	Humanities and Social Sciences	54	20.00%
	Economics and Business Administration	53	19.63%
	Education and Teacher Training	41	15.19%
	Arts and Sports	38	14.07%
	Others	22	8.15%

### 3.2 Variable measurement

All measurement scales used in this study were established instruments, with all variables rated on a 5-point Likert scale, as detailed in Table 2. This study selected student gender, grade level, location, and major category as control variables. Individuals of different genders may exhibit variations in cognitive styles, risk preferences, and creative performance. Controlling for gender can eliminate its potential confounding effects. The depth of education received, knowledge reserves, and university experiences vary among students across different grade levels, which may lead to changes in their creativity levels and development. Even within the Jiangsu-Zhejiang-Shanghai region, subtle differences may still exist among provinces and cities in terms of educational resources, cultural environments, and policy support. Controlling for the location variable can exclude potential regional-level influences. Different academic disciplines vary in knowledge structures, thinking training models, and evaluation criteria, imposing distinct requirements and shaping effects on students’ creativity and innovative behaviors. Controlling for major categories aims to capture variations resulting from disciplinary background differences.

**Table 2:** Reliability and convergent validity.

Variable names	Items	Load value	Cronbach’s *α* coefficient	CR	AVE
Classroom innovation atmosphere (CIA)	Classroom learning creates an atmosphere conducive to smooth communication and interaction among students	0.817	0.924	0.929	0.655
	Teachers encourage students to employ new approaches in solving problems both during and outside class	0.820			
	Classroom learning fosters innovation	0.900			
	Innovative ideas in learning or classroom projects receive support from teachers	0.878			
	Teachers grant us appropriate autonomy to complete tasks	0.867			
	I receive teacher support when encountering learning difficulties	0.759			
	Teachers encourage us to try new learning approaches or solutions	0.581			
Student creativity (SC)	I excel at developing new technologies, processes, and product ideas	0.894	0.934	0.934	0.740
	I frequently conceive creative ideas	0.880			
	I advocate for and promote my ideas to gain others’ approval	0.814			
	I create comprehensive plans and schedules to implement new ideas	0.883			
	I possess innovative capabilities	0.827			
AI technology application (AIT)	When implementing specific learning tasks, I frequently utilize artificial intelligence technologies and equipment, dedicating substantial time to completing tasks in collaboration with AI technologies	0.676	0.895	0.897	0.689
	The application of artificial intelligence technology assists me in achieving personal goals and accomplishments	0.875			
	The application of artificial intelligence technology contributes to enhancing my personal growth	0.885			
	The application of artificial intelligence technology has enhanced my personal capabilities	0.865			
Intrinsic motivation (IM)	I enjoy generating innovative ideas for course projects or academic research	0.867	0.878	0.881	0.650
	I am passionate about improving learning techniques and research methodologies, striving for continuous refinement	0.739			
	I am taking this course primarily because of my strong interest and curiosity in the subject matter itself	0.841			
	I enjoy connecting different concepts or knowledge learned in courses and attempting to use them to generate new insights	0.771			
Self-efficacy (SE)	I believe I excel at proposing novel ideas	0.821	0.894	0.898	0.688
	I have a knack for further supplementing and refining others’ viewpoints	0.873			
	I am confident in my ability to solve problems creatively	0.757			
	I excel at discovering new approaches to problem-solving	0.862			

Classroom Innovation Atmosphere Scale: This study employs the classroom environment scale developed by Fraser (1982). To ensure its applicability, corresponding adjustments were made based on the research context, totaling 7 items.

Student Creativity Scale: This study employs the creativity scale developed by Runco et al. (2001) with appropriate adaptations based on the research context, consisting of five items.

AI Technology Application Scale: This study references the measurement scale developed by Man Tang et al. (2022) and the challenge appraisal subscale from the challenge-threat stress appraisal scale developed by LePine et al. (2016), with appropriate modifications made for the AI context, totaling 4 items.

Intrinsic Motivation Scale: Based on Amabile’s theoretical framework and related pilot studies (Amabile, 1985). This scale’s items were designed to assess students’ level of intrinsic motivation in classroom activities, totaling 4 items.

Self-Efficacy Scale: This study employs the innovative self-efficacy scale developed by Tierney and Farmer (2002), which consists of 4 items designed to assess individuals’ confidence in their success during innovative activities.

### 3.3 Data processing methods

This study is based on a regression analysis framework, utilizing the PROCESS macro program (Model 7) developed by Hayes to analyze mediating, moderating, and moderated mediation effects. The Bootstrap sampling method was employed to derive 95% confidence intervals, testing the significance of indirect and conditional effects to ensure the robustness of the analysis results.

## 4 Results

### 4.1 Common method Bias test

(1) This study employed Harman’s single-factor test method to conduct unrotated principal component analysis on the sample data, forcing the extraction of one factor. The results showed that the first principal component explained 34.492% of the variance, which is below the 40% criterion, indicating that common method bias is not severe.
(2) This study used confirmatory factor analysis to compare the goodness-of-fit between the five-factor baseline model and the single-factor model. The results revealed that the fit indices of the single-factor model (*χ*^2^/df = 12.195, ΔCFI = 0.429, ΔTLI = 0.320, ΔRMSEA = 0.154) were significantly worse than those of the baseline model (*χ*^2^/df = 2.117, ΔCFI = 0.945, ΔTLI = 0.932, ΔRMSEA = 0.049). These results suggest that any potential common method bias in this study did not affect the research findings.

### 4.2 Reliability and validity tests of the questionnaire

This study used SPSS 27.0 software to analyze the reliability and validity of the variables, with the results shown in Table 2.

1. Reliability Analysis. This study selected Cronbach’s alpha coefficient and composite reliability (CR) as evaluation criteria. The test results showed that all variables had Cronbach’s alpha values greater than 0.8, indicating high reliability of the variables.
2. Validity Analysis. This study examined validity from three aspects: content validity, convergent validity, and discriminant validity. Regarding content validity, most measurement items in this study were derived from mature scales both domestically and internationally, demonstrating high content validity. For convergent validity, factor analysis was conducted on the items, with Bartlett’s approximate chi-square of 5,057.439 (*p* < 0.001) and a KMO index of 0.894. Using SPSS 27.0 for factor analysis, five principal factors were extracted, cumulatively explaining 75.185% of the total variance. After applying varimax rotation, all items of the core variables exhibited factor loadings exceeding the threshold of 0.5 on their respective principal factors. The composite reliability (CR) values for each variable’s factor loadings were all greater than 0.7, and the average variance extracted (AVE) values all exceeded 0.5, confirming convergent validity. For discriminant validity, combining Tables 2 and 3, the square roots of the AVE values for all variables were greater than their Pearson correlation coefficients, indicating high discriminant validity.

**Table 3:** Results of discriminant validity, descriptive statistics, and correlation analysis.

Variables	1	2	3	4	5	6	7	8	9
1. Gender	–								
2. Grade level	0.039	–							
3. Location (Province)	0.08	0.004	–						
4. Professional categories	−0.004	0.071	−0.012	–					
5. Classroom innovation atmosphere	−0.051	0.056	−0.016	−0.043	–				
6. Creativity	−0.005	0.116	−0.017	−0.043	0.169^**^	–			
7. AI technology applications	−0.054	−0.025	−0.033	0.002	0.139^*^	0.186^**^	–		
8. Intrinsic motivation	−0.063	0.105	0.037	0.009	0.166^**^	0.585^***^	0.163^**^	–	
9. Self-efficacy	−0.074	0.11	−0.016	0.045	0.180^**^	0.652^***^	0.263^***^	0.651^***^	–
Mean	1.367	2.01	2.03	3.02	4.53	3.927	2.809	3.753	3.732
Standard deviation	0.483	0.974	0.811	1.605	0.608	0.689	1.01	0.616	0.677
AVE square root	–	–	–	–	0.809	0.86	0.83	0.806	0.829

^***^p < 0.001, ^**^p < 0.01, ^*^p < 0.05.

Although within the measurement model certain individual item factor loadings did not meet the recommended threshold of 0.7, these values still exceeded the minimum requirement of 0.5, indicating acceptable explanatory power. The composite reliability and average variance extracted for all variables met the standards, demonstrating high overall reliability and validity of the measurement model. This study concludes that the lower loadings of individual items do not compromise the reliability of the research findings.

### 4.3 Results of descriptive statistics and correlation analysis

This study conducted correlation analysis on the relationships between variables, with results shown in Table 3. The findings indicate that classroom innovation atmosphere is significantly positively correlated with student creativity (*β* = 0.169, *p* < 0.01); classroom innovation atmosphere shows significant positive correlations with both AI technology application and intrinsic motivation (*β* = 0.139, *p* < 0.05; *β* = 0.166, *p* < 0.01); while AI technology application and intrinsic motivation both demonstrate significant positive correlations with creativity (*β* = 0.186, *p* < 0.01; *β* = 0.585, *p* < 0.001). These results provide preliminary support for the research hypotheses.

### 4.4 Hypothesis testing analysis

#### 4.4.1 Main effect testing

The results of the main effect test are presented in Table 5. With creativity as the dependent variable, after introducing classroom innovation atmosphere as the independent variable, the adjusted *R*^2^ value of Model 2 increased from 0.017 to 0.042, indicating a significant improvement in the model’s explanatory power. The regression results show that classroom innovation atmosphere has a significant positive effect on creativity (*β* = 0.182, *p* < 0.01), thus supporting Hypothesis H1.

**Table 5:** Results of the main effect test.

Variables	Model 1	Model 2
Gender	−0.012	0
Grade level	0.085	0.078
Location (Province)	−0.015	−0.013
Field of study	−0.022	−0.019
Classroom innovation atmosphere		0.182^**^
Adj-*R*^2^	0.002	0.024
*R* ^2^	0.017	0.042
*F*-value	1.118	2.33^*^

^**^p < 0.01, ^*^p < 0.05.

This study includes gender, grade, location, and major category as control variables in the analysis. None of these variables had a statistically significant impact on creativity, indicating that within the scope of this study’s sample, demographic characteristics have limited explanatory power for student creativity. This may suggest that, compared to individual background traits, psychological and environmental factors such as classroom innovation atmosphere, intrinsic motivation, and self-efficacy have a more pronounced influence on student creativity. Although the control variables did not show significant effects, they were retained in the model to account for potential confounding influences and to maintain the robustness of the core variable relationships.

#### 4.4.2 Mediation effect test

This study used SPSS 27.0 and employed a stepwise regression method to examine the mediation effects, with results shown in Table 6. The specific procedures were as follows: First, regression analysis was conducted with AI technology application and intrinsic motivation as dependent variables, incorporating control variables and classroom innovation atmosphere. As shown in Models 4 and 6, classroom innovation atmosphere was significantly positively correlated with AI technology application and intrinsic motivation (*β* = 0.231, *p* < 0.05; *β* = 0.161, *p* < 0.01). Second, regression analysis was performed with creativity as the dependent variable, incorporating control variables along with AI technology application and intrinsic motivation. Models 7 and 9 revealed that AI technology application and intrinsic motivation were significantly positively correlated with creativity (*β* = 0.129, *p* < 0.01; *β* = 0.652, *p* < 0.001). Third, Models 8 and 10 demonstrated that when creativity served as the dependent variable and classroom innovation atmosphere was included in the regression model simultaneously with AI technology application and intrinsic motivation, the regression coefficients of classroom innovation atmosphere’s impact on creativity decreased from 0.182 to 0.156 and 0.08, respectively, compared to Model 2, while the regression coefficients of AI technology application and intrinsic motivation remained significant (*β* = 0.116, *p* < 0.01; *β* = 0.639, *p* < 0.001). This indicates that both variables play mediating roles in the process through which classroom innovation atmosphere influences student creativity. For the mediating pathway of AI technology application, classroom innovation atmosphere enhances creativity indirectly by promoting the use of AI technology. Although the indirect effect size of this pathway is relatively small, it still demonstrates that in classrooms with a strong innovation atmosphere, students are more inclined to use AI technology as a tool for exploration and creation. This provides empirical evidence for educators to achieve technology integration in teaching. As for the mediating pathway of intrinsic motivation, the actual indirect effect size is larger. When intrinsic motivation is introduced, the direct effect of classroom innovation atmosphere on creativity weakens, indicating that intrinsic motivation plays a substantial explanatory role in the relationship between “classroom atmosphere-creativity.” One of the core values of fostering an innovation atmosphere lies in stimulating students’ intrinsic learning interest and desire for exploration, which is crucial for cultivating long-term and high-level creativity in students. Thus, Hypotheses H2 and H3 received preliminary support.

**Table 6:** Mediation effect test results.

Variables	AI technology application		Intrinsic motivation		Creativity			
	Model 3	Model 4	Model 5	Model 6	Model 7	Model 8	Model 9	Model 10
Applications of AI technology	−0.107	−0.092	−0.09	−0.079	0.002	0.011	0.047	0.051
Gender	−0.024	−0.033	0.068	0.062	0.088	0.082	0.041	0.039
Grade level	−0.035	−0.033	0.032	0.034	−0.01	−0.009	−0.035	−0.034
Location (Province)	0.002	0.006	0.001	0.004	−0.023	−0.02	−0.023	−0.021
Major		0.231^*^		0.161^**^		0.156^*^		0.08
Classroom innovation atmosphere					0.129^**^	0.116^**^		
Intrinsic motivation							0.652^***^	0.639^***^
Adj-*R*^2^	−0.011	0.005	0.002	0.024	0.034	0.049	0.338	0.341
*R* ^2^	0.004	0.023	0.017	0.042	0.052	0.07	0.351	0.355
*F*-value	0.285	1.266	1.169	2.332^*^	2.897^*^	3.317^**^	28.519^***^	24.174^***^

^***^p < 0.001, ^**^p < 0.01, ^*^p < 0.05.

#### 4.4.3 Moderating effect test

This study examined the moderating effect of self-efficacy through hierarchical regression, with results presented in Table 7. First, to avoid multicollinearity between independent variables and interaction terms, classroom innovation atmosphere and self-efficacy were centered, and their interaction term was calculated. Second, regression analysis was conducted with AI technology application and intrinsic motivation as dependent variables, sequentially introducing control variables, classroom innovation atmosphere, self-efficacy, and their interaction term. Model 11 shows that the interaction term between classroom innovation atmosphere and self-efficacy had a significant positive effect on AI technology application (*β* = 0.339, *p* < 0.05); Model 12 indicates that the interaction term between classroom innovation atmosphere and self-efficacy significantly affected intrinsic motivation (*β* = 0.083, *p* < 0.05). The evaluation of effect sizes for moderation effects revealed that the standardized coefficient for the interaction term between self-efficacy and classroom innovation atmosphere on AI technology application was *β* = 0.339 (*p* < 0.05), while for intrinsic motivation it was *β* = 0.083 (*p* < 0.05). The moderating role of self-efficacy was more pronounced in the pathway of AI technology application—for students with higher self-efficacy, a positive classroom innovation atmosphere more effectively promoted their practical use of AI technologies. Regarding stimulating students’ intrinsic motivation, self-efficacy also played a facilitative role, though with a relatively smaller moderating magnitude. This finding suggests that educators, when leveraging classroom atmosphere to enhance students’ technology application behavior, should particularly focus on strengthening students’ self-efficacy, as the resulting enhancement effect may be more substantial. Therefore, hypotheses H4a and H4b were supported.

**Table 7:** Test results of the moderating effect of self-efficacy.

Variables	AI technology application	Intrinsic motivation
	Model 11	Model 12
CIA	−0.054	−0.023
Gender	−0.079	0.017
Grade level	−0.03	0.038
Location (Province)	−0.001	−0.007
Major	0.217^*^	0.063
SE	0.38^***^	0.583^***^
CIA × SE	0.339^*^	0.083^*^
Adj-*R*^2^	0.076	0.417
*R* ^2^	0.1	0.433
*F*-value	4.152^***^	28.542^***^

^***^p < 0.001, ^*^p < 0.05.

#### 4.4.4 Moderated mediation effect test

This study used Model 7 in the Process plugin to test the moderated mediation effect, with results shown in Table 8. The results indicate that the moderated mediation effect value of AI technology application was 0.0373, with a confidence interval [0.0023, 0.1195] not including 0, thus confirming the existence of moderated mediation. At low levels of the moderating variable, the confidence interval for the mediation effect [−0.0179, 0.0454] included 0; whereas at medium and high levels of the moderating variable, the confidence intervals were [0.0050, 0.0650] and [0.0102, 0.1001] respectively, both excluding 0. This demonstrates that the significance of the mediation effect varies across different levels of the moderating variable. Only when self-efficacy reaches a certain level can classroom innovation atmosphere positively promote creativity through AI technology application. Further conditional process analysis indicates that the indirect promotion effect of classroom innovation atmosphere on creativity through AI technology application is only significant when students’ self-efficacy is at average or higher levels. For students with low self-efficacy, this pathway shows no significant facilitating effect. This suggests that when students lack confidence, even in an innovation-encouraging classroom environment, they are less likely to effectively leverage AI technology to enhance their creativity. Interventions that solely focus on improving classroom settings or introducing technological tools—while neglecting the cultivation of students’ self-belief—may yield very limited results for this group.

**Table 8:** Results of moderated mediation effect test.

Path	Moderator variable level, effect indicator	Path coefficient	Standard error	BootLLCI	BootULCI
CIA-AIT-SC	Low (−1SD)	0.0146	0.0156	−0.0179	0.0454
	Medium (Mean)	0.0298	0.0156	0.0050	0.0650
	High (+1SD)	0.0450	0.0237	0.0102	0.1001
	Difference in indirect effects between high and low levels	0.0304	0.0251	0.0019	0.0974
	Moderated mediation effect index	0.0373	0.0309	0.0023	0.1195
CIA-IM-SC	Low (−1SD)	−0.0885	0.0584	−0.2508	−0.0133
	Medium (Mean)	0.0731	0.0420	−0.0029	0.1652
	High (+1SD)	0.2347	0.0798	0.1397	0.4660
	Difference in indirect effects between high and low levels	0.3231	0.1119	0.2047	0.6574
	Moderated mediation effect index	0.6574	0.1373	0.2512	0.8066

The moderated mediation effect value of intrinsic motivation was 0.6574, with a confidence interval [0.2512, 0.8066] not including 0, thus confirming the existence of moderated mediation. At medium levels of the moderating variable, the confidence interval for the mediation effect was [−0.0029, 0.1652], including 0; whereas at low and high levels of the moderating variable, the confidence intervals were [−0.2508, −0.0133] and [0.1397, 0.4660] respectively, both excluding 0. This demonstrates that the significance of the mediation effect varies across different levels of the moderating variable. Only when self-efficacy reaches a certain level can classroom innovation atmosphere positively promote creativity through intrinsic motivation. In the path of “classroom innovation atmosphere—intrinsic motivation—creativity,” the moderated mediation effect index was larger, and under high self-efficacy conditions, the strength of the indirect effect was greater. This further highlights the crucial role of enhancing students’ self-efficacy in amplifying the positive effects of the classroom environment. Hypotheses H5a and H5b were supported.

#### 4.4.5 Robustness test

Following the research of Lu and White (2014), this study conducted robustness tests by adjusting sample sizes and changing testing methods. Additionally, using the Process 3.3 plugin developed by Hayes (2018), with Bootstrap = 5,000 and a 95% confidence interval, we further examined the mediating effects of AI technology application and intrinsic motivation. The results showed that the indirect effect values of AI technology application and intrinsic motivation between classroom innovation atmosphere and creativity were 0.0267/0.1029 respectively, with corresponding confidence intervals of [0.0034, 0.0598] and [0.0281, 0.1820], neither containing zero. These findings further validate hypotheses H2 and H3.

**Figure 2:**
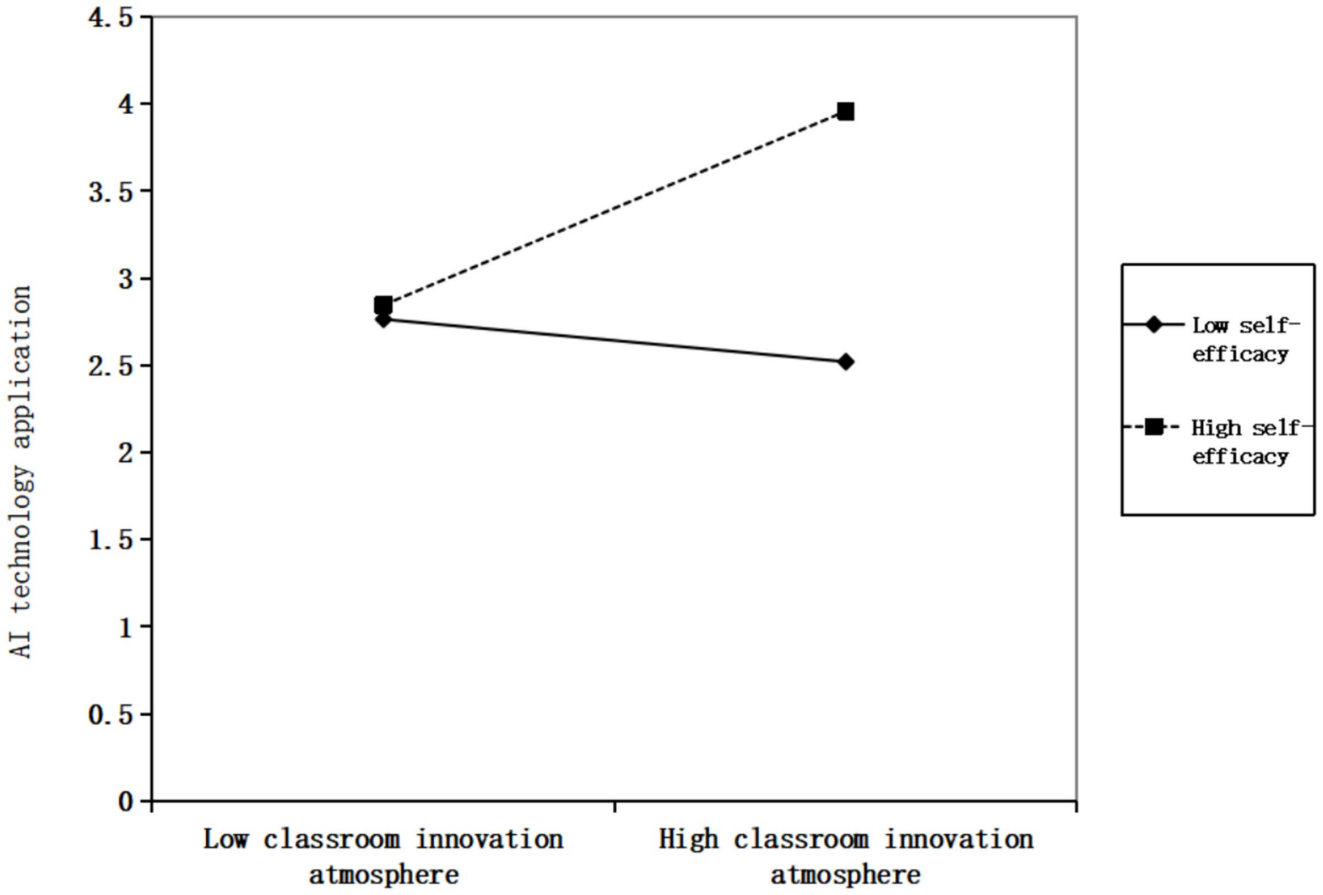
Moderating effect of self-efficacy on the relationship between classroom innovation atmosphere and AI technology application.

**Figure 3:**
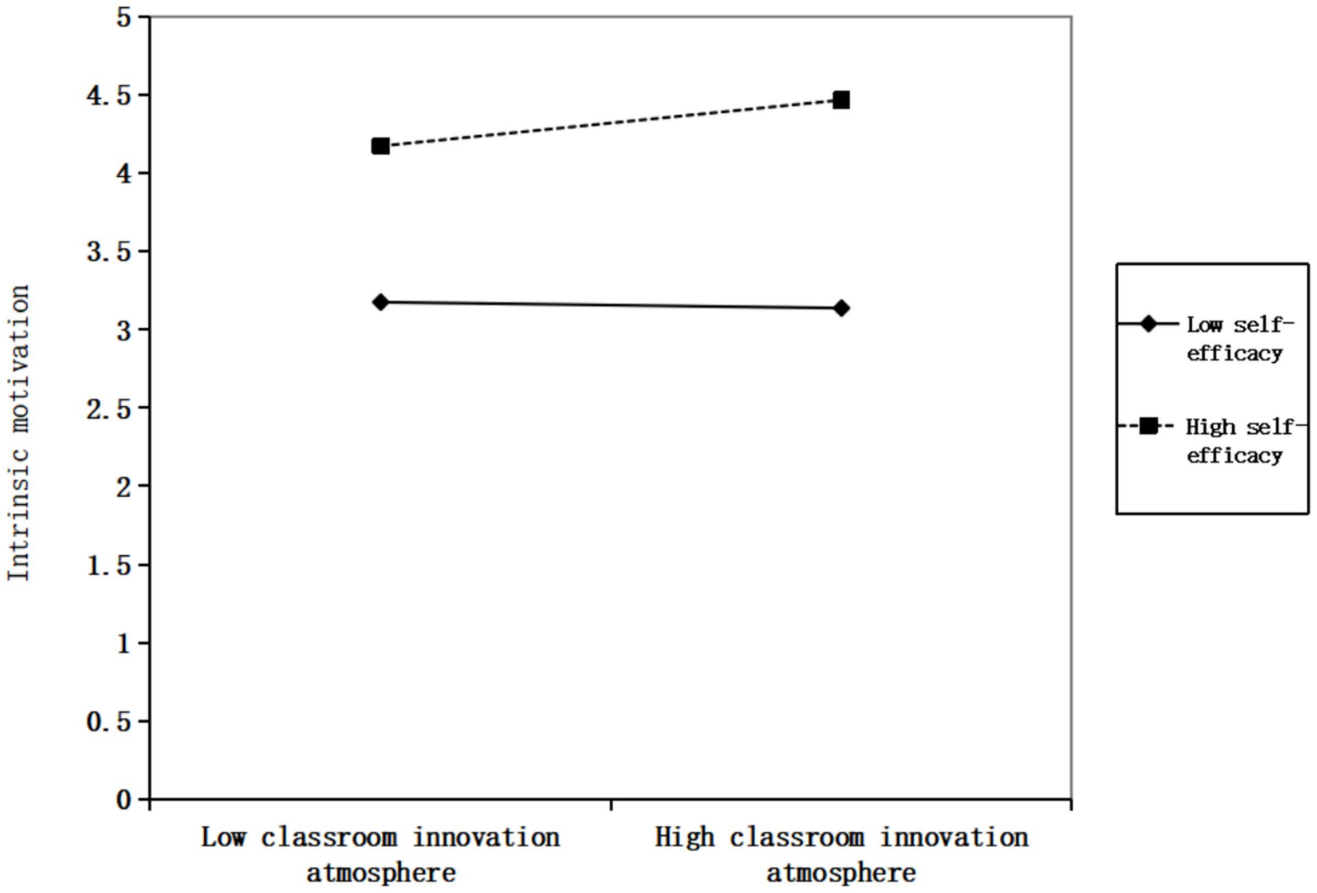
Moderating effect of self-efficacy on the relationship between classroom innovation atmosphere and intrinsic motivation.

This study employed simple slope analysis to further examine the moderating effect of self-efficacy between classroom innovation atmosphere and AI technology application/intrinsic motivation. Figure 2 demonstrates that when self-efficacy is at a high level, classroom innovation atmosphere significantly promotes AI technology application, thereby further validating hypothesis H4a. Figure 3 indicates that when self-efficacy reaches a high level, classroom innovation atmosphere shows significant enhancement effects on intrinsic motivation, thus further confirming hypothesis H4b.

## 5 Discussion

### 5.1 Conclusion

This study explored the mechanism of how classroom innovation atmosphere affects university students’ creativity, yielding the following main conclusions.

This study constructs and validates a moderated mediation model, demonstrating that the classroom innovation atmosphere not only directly enhances student creativity but also operates indirectly through two pathways—promoting AI technology application and fostering intrinsic motivation—with students’ self-efficacy playing a significant positive moderating role in this process. When teachers cultivate an open and inclusive classroom environment, it effectively stimulates students’ creative thinking and enhances their innovation capacity. In such an environment, students can freely express their ideas and actively experiment with new approaches, thereby promoting the development of their creative thinking. This finding breaks through the limitations of previous research that primarily focused on singular pathways of influence. In the relationship between classroom innovation atmosphere and student creativity, the application of AI technology and intrinsic motivation play important mediating roles. This finding highlights the positive effects of classroom innovation atmosphere, which can not only directly impact students’ creativity but also facilitate the effective utilization of AI technology and enhance students’ intrinsic motivation, thereby indirectly improving their creative performance. When designing curricula, educators should consider how to effectively leverage AI technology to enhance students’ creativity. Self-efficacy plays a positive moderating role in the relationship between classroom innovation atmosphere and both AI technology application and intrinsic motivation, further revealing the significance of individual self-efficacy in fostering an innovative classroom atmosphere. When students develop confidence in their abilities, they are more likely to actively engage in innovative classroom environments and fully utilize AI technology for learning and creation. High self-efficacy not only stimulates students’ intrinsic motivation but also strengthens their practical ability to cope with challenges, thereby further promoting creativity enhancement.

This study provides crucial theoretical support and practical insights for educators in fostering students’ creativity, emphasizing the importance of integrating innovation atmosphere, AI technology application, intrinsic motivation, and self-efficacy in classroom teaching. The comprehensive theoretical framework presented analyzes the intrinsic logic of dynamic interactions among these factors, pointing the way for future educational research and practice.

### 5.2 Theoretical contributions

From a theoretical perspective, the primary contribution of this study lies in constructing and validating a moderated multiple mediation model, which dissects the intrinsic mechanism through which classroom innovation atmosphere influences student creativity via the application of AI technology and intrinsic motivation, while also highlighting the enhancing moderating role of self-efficacy. This integrated model addresses a theoretical gap in existing literature regarding the synergistic promotion of creativity formation by “technology usage” and “psychological motivation” in classroom settings. The findings resonate with and extend the core concepts of the Technology Acceptance Model (TAM) and UTAUT. A positive classroom innovation atmosphere effectively creates facilitating conditions emphasized in TAM, lowering students’ barriers to adopting AI technology and reducing perceived risks, which aligns with the construct of perceived ease of use. Furthermore, this study reveals that in innovative educational contexts, technology adoption is not merely driven by utilitarian evaluations but also by exploratory interest and innovation-driven proactive actions. Social cognitive theory, with its emphasis on the interaction between individual agency and environmental factors, provides deeper insights into this phenomenon.

This study offers empirical support for the relationship between classroom innovative atmosphere and student creativity. The research proposes that classroom innovative atmosphere is a key factor in enhancing students’ creativity, further demonstrating the importance of environmental factors in education (Wang et al., 2023). The classroom innovative atmosphere encompasses not only teachers’ instructional methods but also student interaction and collaboration. A dynamic classroom environment encourages students to boldly experiment with new ideas and helps cultivate their creative thinking. This finding not only provides empirical evidence for educational theory but also suggests new directions for future research.

The application of AI technology and intrinsic motivation play mediating roles in the relationship between classroom innovative atmosphere and creativity. With rapid technological advancements, educators face the challenge of effectively utilizing modern AI technology to enhance students’ learning capabilities. Applying AI technology can not only enrich learning resources but also stimulate students’ intrinsic motivation, making them more proactive in engaging in creative activities (Deci and Ryan, 2000). In the process of using AI technology to promote students’ innovative abilities, educators must not overlook the importance of humanistic care. Education is not merely about imparting knowledge but also about fostering students’ holistic development. Teachers should encourage students to think boldly, thereby igniting their intrinsic motivation and promoting creativity cultivation. This further highlights that intrinsic motivation is a crucial element in driving creativity (Ballesteros et al., 2023).

This study also explores the moderating role of self-efficacy in the relationship between classroom innovation atmosphere, AI technology application, and intrinsic motivation. The results once again confirm that self-efficacy plays a crucial role in the learning process (Chiu et al., 2023). Amid the growing rise of AI in educational applications, this study clarifies the mediating role of instrumental AI technology as an enabling pathway in the relationship between innovative atmosphere and creativity. It addresses the practical challenge of how technology integrates into educational ecosystems to drive innovation by incorporating self-efficacy into the model. The research reveals self-efficacy as a key individual cognitive resource that enhances the environmental push for technology adoption while strengthening the stimulating effect of the environment on intrinsic motivation. This expands the application scope of social cognitive theory in digital educational settings. Enhancing self-efficacy not only boosts students’ learning confidence but also motivates them to actively engage in innovative practices. These findings suggest that educators should pay attention to students’ self-efficacy when designing curricula, employing practical methods to strengthen their confidence. By providing encouragement and helping students recognize their progress, educators can further facilitate the development of their creativity. This study reveals an orderly chain of effects—"environmental support, technology empowerment, motivation stimulation, and belief regulation”—establishing a more comprehensive and dynamic theoretical framework for understanding the cultivation mechanisms of student creativity. These findings not only offer theoretical guidance for educators in fostering students’ creativity but also open up new directions for future research. Educators can leverage this theoretical framework to develop more targeted teaching strategies, thereby promoting the cultivation of students’ innovative abilities and the enhancement of their comprehensive competencies.

### 5.3 Practical implications

Based on the findings of this study, the following recommendations are proposed for educational practices in higher education institutions to more effectively cultivate students’ creativity.

Educators should strive to build an innovative learning environment supported by AI technology. Teachers should incorporate data analysis tools into curriculum design and utilize intelligent literature management software to assist in research, freeing students from the cognitive burden of complex information processing. For example, “human-machine collaboration” research projects can be designed, explicitly requiring students to use these tools for tasks such as data cleaning, visualization, or literature tracing. The saved effort can then be directed toward proposing innovative research hypotheses, deeply interpreting analytical results, and explaining their theoretical and practical significance. Course objectives should clearly define the supportive role of AI, emphasizing its value in extending rather than replacing human intellect, ensuring that students hone their critical thinking and original conceptual abilities while adopting such technologies.

To ensure the healthy development of technology applications, schools should introduce concise guidelines for the use of artificial intelligence. These guidelines must first establish clear boundaries for academic integrity, prohibiting the use of AI for automated writing, data manipulation, and similar misconduct, while incorporating such violations into academic integrity policies. More importantly, they should guide students in developing responsible usage habits. When using AI for data processing tasks, students must specify the tools employed, the analytical procedures followed, and their interpretation and verification of the results in their assignments or reports, thereby enhancing scientific rigor and transparency in research. Institutions may provide standardized citation and disclosure templates to facilitate student compliance and faculty verification.

Student self-efficacy is a key factor in facilitating environmental support and technological innovation. In the teaching process, it is necessary to incorporate strategic methods to enhance technological confidence. Before introducing new AI software, teachers should provide micro-training tutorials with clear tasks and steps, rather than complex theoretical explanations, thereby reducing students’ technical anxiety at the initial stage. When assigning complex projects, the task segmentation method should be employed—breaking large projects into smaller components—and timely feedback should be given at each stage, enabling students to visibly recognize their progress and skill development. Teachers must pay special attention to openly acknowledging students’ exploratory spirit when using tools to solve practical problems. This process-oriented praise is particularly effective in building long-term, stable technological confidence.

As the trend of artificial intelligence becoming ubiquitous grows increasingly evident, the evaluation of students’ creativity must also keep pace with the times. The assessment system should shift from a singular focus on verifying outcomes to a comprehensive approach incorporating process-based portfolios. Schools should encourage students to submit assignments that include complete documentation of their AI utilization workflows—such as screenshots of key data preprocessing steps, comparisons of analytical results with different parameters and reasoning behind selections, as well as independent conclusions and innovative inferences derived from AI outputs. Evaluation criteria must explicitly establish a “depth of thinking empowered by technology” dimension, emphasizing how students leverage tools to expand cognitive horizons and what distinctive integration, critical analysis, and creative achievements they accomplish on this foundation.

The support of school administrators is the backbone for creating a sustainable innovation ecosystem. It is proposed that the school consolidate its current software resources and establish a unified AI platform for teaching tools, providing students with licensed software and stable technical assistance. Additionally, the school should initiate an “AI-empowered Teaching Innovation Seed Fund” to encourage teachers to apply for small-scale reform projects that deeply integrate specific AI tools into teaching modules, while funding the transformation of successful experiences into shareable teaching case studies. Regularly hosting “Human-Machine Collaborative Innovation Project Exhibitions” to showcase outstanding student works that utilize AI to solve real-world problems will foster a campus culture that actively embraces technology for exploration and creation.

Ultimately, educators should adopt a long-term vision to cultivate a continuously improving ecosystem of innovation. A positive, secure, and inspiring classroom environment serves as fertile ground for creativity to thrive. Thoughtful integration of AI technology can spark students’ exploratory drive and sense of competence. In teaching practices, it is crucial to emphasize the synergistic effects of intrinsic motivation, self-efficacy, and technological empowerment, helping students not only find joy in acquiring knowledge but also build self-worth through creative exploration. Creating an innovative classroom atmosphere and nurturing creativity cannot be achieved overnight—it is an evolutionary process requiring continuous evaluation and dynamic adjustment. Educators must develop keen insight and reflective capacity, conducting systematic analysis of teaching strategies, student feedback, and innovation outcomes at regular intervals. By promptly optimizing implementation pathways to align with students’ developmental needs and external environmental shifts, educators can guide students into deeper exploration and autonomous innovation. This multi-layered, sustained systematic approach is essential for cultivating highly creative talent reserves for society.

### 5.4 Research limitations and future prospects

This study has the following limitations. Although the sample was drawn from universities in the Jiangsu-Zhejiang-Shanghai region and possesses certain representativeness, it may still not fully reflect the developmental landscape of student creativity nationwide. Future research could consider expanding the sample scope to include more universities from diverse regions and types, thereby enhancing the validity of the findings. The development of creativity is an ongoing process that may be influenced by multiple factors. Future studies should also examine the long-term impact of classroom innovation atmosphere. This study primarily constructs a model based on social cognitive theory. Future research could introduce and compare other theoretical frameworks, integrating the core variables of the technology acceptance model with the psychological motivation variables of this study to create a more predictive unified model. Alternatively, by leveraging the computational creativity framework, specific characteristics of student-AI collaborative creations could be analyzed, providing objective metrics for assessing creativity. Additionally, The impact mechanisms of generative AI technology on classroom innovation atmosphere and student creativity may differ from those of instrumental AI technology, the primary focus of this study, warranting further validation in future research. This study has certain limitations in the selection of control variables. Although we included common demographic variables such as gender, grade level, location, and major category as control variables, none showed significant influence in the model nor substantially improved the model’s explanatory power. Future research could consider introducing more contextually relevant control variables, such as course type or discipline-specific intrinsic constraints on creativity. These variables may more directly affect students’ behavior and cognitive processes in innovative environments, thereby facilitating a more precise analysis of the mechanisms linking classroom innovation atmosphere and student creativity. While this study provides some theoretical foundations and practical guidance regarding the influence of classroom innovation atmosphere on student creativity, subsequent research needs to improve in areas such as sample diversity and methodological richness to more deeply explore the mechanisms through which classroom innovation atmosphere affects student creativity.

## Data Availability

The raw data supporting the conclusions of this article will be made available by the authors, without undue reservation.
